# Nintendo Switch Joy-Cons’ Infrared Motion Camera Sensor for Training Manual Dexterity in People with Multiple Sclerosis: A Randomized Controlled Trial

**DOI:** 10.3390/jcm11123261

**Published:** 2022-06-07

**Authors:** Alicia Cuesta-Gómez, Paloma Martín-Díaz, Patricia Sánchez-Herrera Baeza, Alicia Martínez-Medina, Carmen Ortiz-Comino, Roberto Cano-de-la-Cuerda

**Affiliations:** 1 Department of Physical Therapy, Occupational Therapy, Physical Medicine and Rehabilitation, Faculty of Health Sciences, Rey Juan Carlos University, 28008 Madrid, Spain; alicia.cuesta@urjc.es (A.C.-G.); roberto.cano@urjc.es (R.C.-d.-l.-C.); 2 International PhD School, Rey Juan Carlos University, 28008 Madrid, Spain; p.martin.2020@alumnos.urjc.es; 3 Asociación de Esclerosis Múltiple de Toledo (ADEMTO), 45007 Toledo, Spain; ademtocf@gmail.com (A.M.-M.); ademtocu1@gmail.com (C.O.-C.)

**Keywords:** multiple sclerosis, upper limb, virtual reality, video games, Joy-Cons, Nintendo Switch, manual dexterity, infrared

## Abstract

Background: The Nintendo Switch^®^ (NS) is the ninth video game console developed by Nintendo^®^. Joy-Cons^®^ are the primary game controllers for the NS^®^ video game console, and they have an infrared motion camera sensor that allows capturing the patient’s hands without the need to place sensors or devices on the body. The primary aim of the present study was to evaluate the effects of the NS^®^, combined with a conventional intervention, for improving upper limb (UL) grip muscle strength, coordination, speed of movements, fine and gross dexterity, functionality, quality of life, and executive function in multiple sclerosis (MS) patients. Furthermore, we sought to assess satisfaction and compliance levels. Methods: A single-blinded, randomized clinical trial was conducted. The sample was randomized into two groups: an experimental group who received treatment based on Dr Kawashima’s Brain Training^®^ for the NS^®^ (20 min) plus conventional rehabilitation (40 min), and a control group who received the same conventional rehabilitation (60 min) for the ULs. Both groups received two 60 min sessions per week over an eight-week period. Grip strength, the Box and Blocks Test (BBT), the Nine Hole Peg Test (NHPT), the QuickDASH, the Multiple Sclerosis Impact Scale (MSIS-29), the Trail Making Test (TMT), and the Stroop Color and Word Test (SCWT) were used pre- and post-treatment. Side effects and attendance rates were also recorded. Results: Intragroup analysis showed significant improvements for the experimental group in the post-treatment assessments for grip strength in the more affected side (*p* = 0.033), the BBT for the more (*p* = 0.030) and the less affected side (*p* = 0.022), the TMT (A section) (*p* = 0.012), and the QuickDASH (*p* = 0.017). No differences were observed for the control group in intragroup analysis, but they were observed in the NHPT for the more affected side (*p* = 0.012). The intergroup analysis did not show differences between both groups. Conclusions: Our results show that an eight-week experimental protocol, after using Dr Kawashima’s Brain Training^®^ and the right-side Joy-Con controller for the NS^®^, combined with a conventional intervention, showed improvements in grip strength, coordination, fine and gross motor function, executive functions, and upper limb functionality in the experimental group. However, no differences were observed when both groups were compared in the intergroup analysis. The addition of Brain Training^®^ for the NS^®^ for the upper limb rehabilitation did not show side effects and was rated with a high satisfaction and excellent compliance in people with MS. Trial registration: This randomized controlled trial has been registered at ClinicalTrials Identifier: NCT04171908, November 2019.

## 1. Introduction

Multiple sclerosis (MS) is a chronic immune-mediated inflammatory demyelinating illness of the central nervous system of unknown etiology and multifactorial origin [1,2]. MS is the cause of disability in younger adults. Among these disabilities, dexterity and activities of daily living (ADL) limitations on the upper limbs (ULs) represent one of the most common problems in patients with MS, usually manifesting as weakness or ataxia. Johansson et al. [3] reported that up to 76% of MS patients present UL impairment, and that in at least 50% of patients, the severity of the dysfunction was moderate. Kamm et al. [4] and Choi et al. [5] reported that after 15 years of disease evolution, the majority of MS patients report hand dysfunction, so patients show compensations or decreased UL functions [4,5], although its importance may be under-recognized relative to walking impairment, which is the hallmark symptom of MS [6].

At present, MS rehabilitation includes technology such as virtual reality (VR), as a complement to conventional therapy programs for hand dexterity, achieving a higher treatment intensity at a sustainable cost [7]. VR through video games presents benefits in the general population such as improvements in overall mobility, eye–hand coordination, space–time organization, and attention and concentration, and benefits in terms of the speed of decision making, memory, social contact when playing in groups, spontaneity, and originality [7,8]. However, few studies exist on the effects that VR has on the manual dexterity of patients with MS.

Serious games are defined as games designed for a primary purpose other than that of pure entertainment, and which promote learning and behavior changes. In the MS context, new low-cost markerless devices have emerged [8,9,10] combined with serious games for rehabilitation aims. However, not all clinical settings or hospitals have these technologies due to acquisition or marketing problems. The Nintendo Switch (NS)^®^ is the ninth video game console developed by Nintendo^®^. It was unveiled in October 2016 and released worldwide in March 2017. Joy-Cons^®^ are the primary game controllers for the NS^®^ video game console. The NS Joy-Cons’^®^ infrared motion camera sensor allows capturing the movement of the patient’s hands without the need to place sensors or devices on the body. Through this device, the patient could be prompted to perform movements and functional tasks in a virtual and encouraging environment. This system presents important advantages over other motion capture systems, for example, its portability, ease of use, commercial availability, relatively low cost, and non-invasive nature [8]. However, there is no previous evidence to support the therapeutic use of the NS^®^ in the treatment of UL motor disorders in people with MS. Moreover, as exercise has the potential to help both motor and nonmotor aspects, there is a need to corroborate this hypothesis in terms of cognitive function in MS patients through the Nintendo Switch (NS).

The primary aim of the present randomized clinical trial was to evaluate the effects of the NS^®^, through the Dr Kawashima’s Brain Training^®^ video game combined with a conventional intervention, for improving UL grip muscle strength, coordination, speed of movements, fine and gross dexterity, functionality, quality of life, and executive functions, compared to a control group. Furthermore, we sought to assess satisfaction and compliance levels in MS patients. Our initial hypothesis was that an experimental protocol system using the NS^®^ and based on a commercial video game could improve UL grip muscle strength, coordination, speed of movements, fine and gross dexterity, functionality, and executive functions.

## 2. Materials and Methods

### 2.1. Design

A single-blinded, parallel-group, randomized clinical trial (RCT) was conducted (NCT04171908) following the Consolidated Standards of Reporting Trials (CONSORT) guidelines. Non-probabilistic sampling of consecutive cases was used. The sample was randomized, after using QuickCalcs GraphPad Software, into a control group (CG) who received conventional rehabilitation treatment and an experimental group (EG) who received treatment with the NS^®^ through the Dr Kawashima’s Brain Training^®^ video game combined with their conventional intervention sessions. All interventions were performed at the Madrid Association of Multiple Sclerosis (Madrid, Spain) and Toledo Association of Multiple Sclerosis (Toledo, Spain).

This study was approved by the local ethics committee. Informed consent was obtained from all participants included in this study.

### 2.2. Participants

The study inclusion criteria were as follows: a diagnosis of MS according to the McDonald criteria; a score of between 3.5 and 7.5 on the Kurtzke Expanded Disability Status Scale (EDSS); stable medical treatment during at least the six months prior to the intervention; muscle tone in the upper limbs not greater than 2 points on the modified Ashworth Scale; a score of 4 points or less in the “Pyramidal Function” section of the EDSS functional scale; ability to understand instructions; a score of 24 points or more in the Mini-Mental Test.

The exclusion criteria were: a diagnosis of another neurological illness or musculoskeletal disorder different to MS; a diagnosis of a cardiovascular, respiratory, or metabolic illness or other conditions which may interfere with the study; suffering a flare-up or hospitalization in the last three months prior to commencement of the assessment protocol or during the process of the therapeutic intervention; receiving a cycle of steroids, either intravenously or orally, six months prior to the commencement of the assessment protocol and within the study period of the intervention; receiving treatment with botulinum toxin in the six months prior to the beginning of the study; and the presence of visual disorders not corrected by optical devices.

### 2.3. Intervention

All patients received the intervention between March and December of 2021 with the same time period of treatment, with a total of 16 sessions.

The control group received a conventional UL intervention by an occupational therapist based on conventional motor rehabilitation therapy (60 min, 2 sessions per week over an eight-week period) based on shoulder, elbow, wrist, and finger mobilization, strengthening of UL extensor muscles, and stretching exercises for UL flexor muscles, with functional and dexterity tasks (i.e., reaching movements, dexterity, and grasping and pincer grasp movements using objects of daily living, such as coins, keys, balls, cups, and plates) [11,12].

The experimental group used Dr Kawashima’s Brain Training^®^ for the NS^®^ (20 min, 2 sessions per week over an eight-week period). A time of 10 min was designated for each upper limb each session. None of the patients had previous experience with this device and with the Dr Kawashima’s video game. Additionally, all patients in the experimental group received the same conventional UL intervention by the same occupational therapist (40 min, 2 sessions per week over an eight-week period).

Joy-Cons^®^ consist of two individual units, each containing an analog stick and an array of buttons. They can be used while attached to the main NS^®^ console unit, or detached and used wirelessly. When detached, a pair of Joy-Cons can be used by a single player or divided between two as individual controllers. Each Joy-Con contains an accelerometer and gyroscope, which can be used for motion tracking. The right-side Joy-Con controller for the NS^®^ includes a motion infrared sensor. This sensor can read how far away objects are and can even detect shapes, and it is capable of differentiating hand movements, including finger and hand shapes such as Rock-Paper-Scissors or finger counting, which allows the user to practice interesting functional exercises for hand dexterity and executive function training.

The experimental protocol was based on the Dr Kawashima’s Brain Training^®^ video game for the NS^®^, designed by Ryuta Kawashima. It was published by Nintendo^®^ for the NS^®^ in December 2019 in Japan and in January 2020 in Europe and Australia. This game is called “Brain Age” in North America. In our study, all patients played all games for both upper limbs in this sequence in all sessions (see Supplementary Materials):

Flag Raising: Players have to memorize and copy the shoulder movements of a virtual character on screen that moves a flag with their upper limb in multiple directions. The aim of the game is for patients to memorize these shoulder movements (flexion, extension, horizontal abduction or adduction) and to reproduce them in the same order as fast as possible.

Birdwatching: in this game, the subject is focused on counting birds, which appear on screen at different speeds, pressing a Joy-Con’s button with the thumb.

Finger Drills: the subject is asked to make 64 hand and finger shapes as fast as possible (open or close hand, thumbs up, different finger positions, etc.).

Finger Calculations: the subject is asked to perform mathematical calculations as fast as possible with their fingers that are captured by the IR motion camera.

Rock-Paper-Scissors game: at random intervals, subjects are asked to win or lose in the Rock-Paper-Scissors game, capturing their movements with the infrared sensor.

### 2.4. Measures

All assessments were performed by the same blinded raters trained in the use of the measures. The following outcome measures were used pre- and post-treatment:

Grip strength. A Jamar^®^ hydraulic hand dynamometer was used to measure grip strength. This dynamometer offers accurate and repeatable grip strength readings scaled in pounds and kilograms. All the patients performed three grip movements, and the mean values were recorded. The data for the less and more affected sides were recorded in kilograms. The Jamar^®^ hydraulic hand dynamometer is one of the most commonly used objective tools to assess grip strength and is considered a device of excellent reliability, sensitivity, and ease of use. It is recommended by the American Society of Hand Therapists and by the Brazilian Society of Hand Therapists [13].

The Box and Blocks Test (BBT) was performed to measure unilateral gross manual dexterity on both the less and the more affected side. The BBT consists of moving the maximum number of blocks from one compartment of a box to another, one by one, within one minute. The BBT is a quick, simple, and reliable measurement of manual dexterity. The BBT administration procedure is standardized, and its validity has been shown in elderly subjects with upper limb disability [14,15].

The Nine Hole Peg Test (NHPT) was used. It is a hand function test, which consists of a plastic peg board (25.0 cm × 12.7 cm × 2.3 cm) with nine holes (2.54 cm between the holes) and nine pegs (3.2 cm long, 0.64 cm wide). The participant has to put the nine pegs in the peg board as fast as possible, one at a time with one hand only, and then remove them again. The test is performed two times per hand, with the non-affected hand first. The time it took to fulfill the second trial was used for the analysis [16].

QuickDASH. The QuickDASH is a shortened version of the DASH outcome measure. Instead of 30 items, the QuickDASH uses 11 items (scored 1–5) to measure physical function and symptoms in people with any or multiple disorders of the upper limbs. The QuickDASH is a widely used reference of self-reported disability for MS patients [6]. Scale scores are calculated, ranging from 0 (no disability) to 100 (most severe disability).

Multiple Sclerosis Impact Scale (MSIS-29). This scale is a specific instrument that allows for assessing the physical and psychological well-being of subjects with MS. It is made up of 29 questions divided into two components: a physical magnitude comprising the first 20 questions, and a psychological magnitude with the last 9 questions. The answers are scored on a Likert scale from 1 to 5, with a maximum of 100 points in the physical part and 45 points in the psychological evaluation [17]. The results are interpreted as a percentage measure. The MSIS-29 has demonstrated its validity and suitability for the evaluation of people with MS, compared to other established measures [18]. It is considered a reliable method that assesses quality of life within the field of MS [17]. The MSIS-29 scale is among the 20 specific scales validated for the measurement of quality of life in the context of MS and is among the 3 most commonly used according to a number of articles published in this regard.

The Trail Making Test (TMT) is a neuropsychological test of visual attention and task switching composed of two sections. Both parts of the Trail Making Test consist of 25 circles distributed over a sheet of paper. In Part A, the circles are numbered 1–25, and the patient should draw lines to connect the numbers in ascending order. In Part B, the circles include both numbers (1–3) and letters (A–L); as in Part A, the patient draws lines to connect the circles in an ascending pattern, but with the added task of alternating between the numbers and letters (1-A-2-B-3-C, etc.). The patient should be instructed to connect the circles as quickly as possible, without lifting the pen or pencil from the paper. If the patient makes an error, the rater must point it out immediately and allow the patient to correct it. Errors affect the patient’s score only in that the correction of errors is included in the completion time for the task. It is unnecessary to continue the test if the patient has not completed both parts after five minutes have elapsed. Results for both the TMT-A and B are reported as the number of seconds required to complete the task; therefore, higher scores reveal greater impairment [19].

The Stroop Color and Word Test (SCWT) is a neuropsychological test extensively used to assess the ability to inhibit cognitive interference that occurs when the processing of a specific stimulus feature impedes the simultaneous processing of a second stimulus attribute [20]. In the most common version of the SCWT, which was originally proposed by Stroop in 1935, subjects are required to read three different tables as fast as possible. Two of them represent the “congruous condition” in which participants are required to read names of colors (henceforth referred to as color-words) printed in black ink (W) and name different color patches (C). Conversely, in the third table, named color-word (CW) condition, color-words are printed in an inconsistent color ink (for instance, the word “red” is printed in green ink). Thus, in this incongruent condition, participants are required to name the color of the ink instead of reading the word. In other words, the participants are required to perform a less automated task (i.e., naming ink color) while inhibiting the interference arising from a more automated task (i.e., reading the word). While the SCWT is widely used to measure the ability to inhibit cognitive interference, the previous literature also reports its application to measure other cognitive functions such as attention, processing speed, cognitive flexibility, and working memory. Thus, it may be possible to use the SCWT to measure multiple cognitive functions [20].

Satisfaction. The Client Satisfaction Questionnaire (CSQ-8) was used to evaluate the satisfaction of health service users for both groups. This is a self-administered post-treatment questionnaire, composed of eight items that evaluate the level of satisfaction regarding the care and quality of the service received and the level of fulfillment of the patient’s expectations regarding the treatment administered. The total score of the questionnaire is 32 points, with higher values meaning higher satisfaction with the treatment received [21,22]. The result is calculated as a percentage measure. In addition, patients completed a satisfaction questionnaire experimental related to the NS^®^ treatment program. It was designed by the research group based on previous publications on using video games in MS [23]. The questionnaire was composed of 18 items that assess the degree of satisfaction in the following dimensions: technical quality and operation of the equipment (4 items); ease of the video game to be played even in disadvantageous conditions (5 items); program compliance and satisfaction in relation to the treatment performed and its applicability (7 items); general degree of satisfaction or complacency (2 items). The answers of this questionnaire are established on a five-point Likert scale, from not satisfied (=1) to very satisfied (=5), with alternative directionality to reduce stereotyped responses. Regarding the interpretation of the results of the surveys, the total score was calculated as a percentage measure.

NASA Task Load Index (NASA) scale. This scale is a subjective, multidimensional assessment tool that rates perceived workload in order to assess a task or other aspects of performance. The NASA scale is divided into six subjective subscales (Mental Demand, Physical Demand, Temporal Demand, Performance, Effort, and Frustration). They are rated for each task within a 100-point range with 5-point steps. These ratings are then combined with the Task Load Index (%) [24].

Additionally, we recorded side effects and the attendance rate (%) for therapy sessions (compliance).

### 2.5. Statistical Analysis

Statistical analysis was performed using the SPSS statistical software system (SPSS Inc., Chicago, IL, USA, version 27.0). A descriptive analysis was carried out of all variables. The Shapiro–Wilk test was used to screen all data for the normality of the distribution. The hypothesis that the variables did not have a normal distribution was accepted, due to the results of the test, the verification of the histograms of each variable, and the size of the sample. The Wilcoxon test for related samples was used to compare variables. Statistical analysis was performed with a 95% confidence level, and significant values were considered as *p* < 0.05. Additionally, the Mann–Whitney test for non-related samples was used to compare variables, where significant values were considered as *p* < 0.05.

## 3. Results

**Figure 1:**
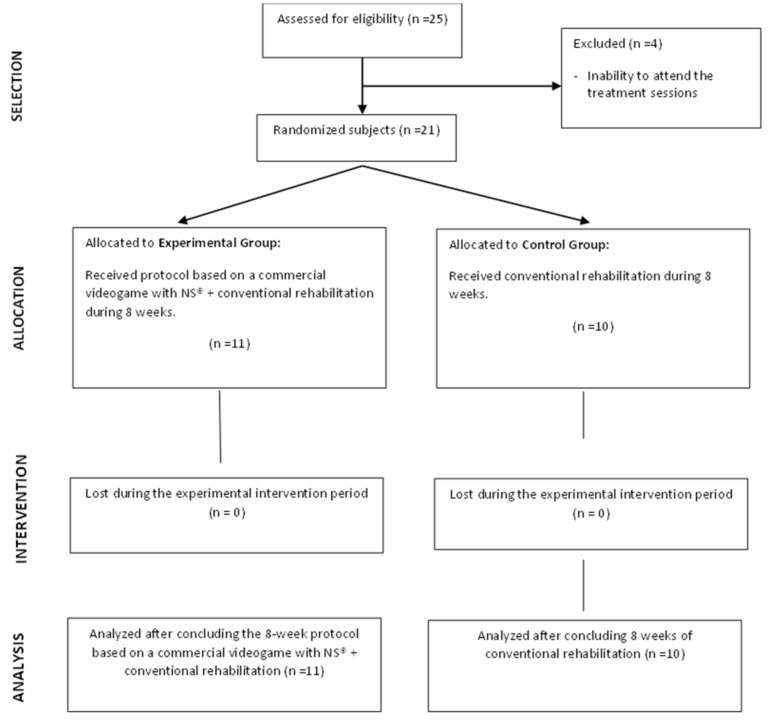
Flow diagram.

The initial sample consisted of 25 patients. However, four of them were excluded due to an inability to attend the treatment sessions. The final sample consisted of 21 patients (9 men and 12 women) that were randomly assigned into two groups by means of the QuickCalcs application of GraphPad Software^®^ (GraphPad Inc, San Diego, CA, USA): 11 were assigned to the experimental group, while 10 were assigned to the control group (Figure 1). Table 1 shows the socio-demographic data of the sample.

The within-group statistical analysis for the experimental group showed significant improvements in the grip strength for the more affected side (*p* = 0.033); the BBT for the more (*p* = 0.030) and the less affected side (*p* = 0.022); the TMT-A (*p* = 0.012); and the QuickDASH (*p* = 0.017). All these results indicate improvements in these scores after the experimental treatment. In the within-group statistical analysis for the control group, statistically significant differences were observed only in the NHPT for the more affected side (*p* = 0.012). Patients showed longer times when performing the NHPT in the post-treatment measurements, which indicated poorer results (Table 2).

According to the intergroup statistical analysis, no significant differences were observed for the clinical variables analyzed in both groups (Table 3).

In addition, no adverse side effects were observed, with 21.62 ± 10.55 points for the NASA Task Load Index on a 100-point scale. The patients for both groups showed a high degree of satisfaction measured through the CSQ-8. The experimental group obtained a mean of 94.70 ± 1.43 points, and the control group obtained a mean of 93.18 ± 2.55 points. Regarding the scale of satisfaction with the technology, the experimental group obtained an average of 91.04 ± 2.56, indicating that the patients were very satisfied with the virtual treatment. The best rated items were: technical quality and operation of the equipment; program compliance and satisfaction in relation to the treatment performed and its applicability and general degree of satisfaction. The attendance rate for the interventions was excellent (100%) in both groups.

## 4. Discussion

To our best knowledge, this is the first single-blinded RCT that used the NS^®^ as a UL rehabilitation tool in people with MS. The aim of the present study was to investigate the effects of the NS^®^ through the Dr Kawashima’s Brain Training^®^ video game, as a coadjutant treatment for improving grip strength, manual dexterity, functionality, quality of life, and multiple cognitive functions in people with MS, compared to a control group which received a conventional rehabilitation for the ULs. Our results show that an eight-week period of using the NS^®^ for UL treatment showed improvements in grip strength, coordination, fine and gross motor functions, executive functions, and upper limb functionality within the experimental group. No intergroup differences were observed when both groups were compared. Furthermore, a high satisfaction and an excellent attendance were achieved for both groups, and the experimental protocol did not show adverse side effects, so this technology could be used to increase the level of satisfaction and compliance of traditional interventions focused on UL treatment in people with MS.

Few studies have been conducted for UL rehabilitation in MS patients with technology. Previously, Cuesta et al. [8] evaluated the effectiveness of the Leap Motion Controller and serious games specifically designed for UL treatment in 30 people with MS. Their experimental protocol was based on two 60 min sessions per week over a ten-week period of conventional motor rehabilitation therapy (45 min) plus the Leap Motion Controller (15 min) with a total of 20 sessions. Waliño-Paniagua et al. [7] assessed the effectiveness of occupational therapy plus semi-immersive virtual reality via a webcam for UL rehabilitation in 16 MS patients. They used 20 sessions of occupational therapy, lasting 30 min, twice weekly, plus 20 min of VR via a webcam. Jonsdottir et al. [25] studied the feasibility and efficacy of a serious game approach, using the Kinect combined with conventional rehabilitation, to supervised UL rehabilitation in 18 MS patients, with 12 sessions, 3–5 times per week, lasting 45 min. Finally, Jonsdottir et al. [26] studied the feasibility of a serious game platform using the Kinect vs. the Nintendo Wii combined with conventional rehabilitation for UL treatment in 16 people with MS with a protocol of 12 sessions, 40 min per session, 4–5 sessions per week. Almost all technologies previously used failed to address the complexity of UL movements in a rehabilitation context, due to their limited ability to recognize hand and finger movements. Even in the research conducted by Cuesta et al. [8], the serious games used for the Leap Motion Controller are not available for download or general purchase. This was one of the main reasons to consider the NS^®^ and the Dr Kawashima’s Brain Training^®^ video game for this research. Our protocol was based on 16 sessions of 20 min, twice weekly over an eight-week period. It must be noted that 10 min was designated for each upper limb each session and that all patients received a previous conventional UL intervention for 40 min, meaning the intragroup results must be interpreted as a combination of both types of therapies.

Statistical analysis did not show significant results for any of the variables included in the intergroup analysis. Although our experimental protocol was focused on the ULs, and more specifically on hand and finger movements, the lack of differences between groups may be due to the treatment time period and the risk of fatigue that could limit a more intensive intra-session training and an extended time of treatment in terms of the number of sessions.

However, clinical improvements were observed in several outcomes for both groups. It should be taken into account that all patients recruited in our research presented a moderate state of the disease, so the conventional rehabilitation and the protocol based on Dr Kawashima’s Brain Training^®^ for the NS^®^ over an eight-week period could maintain the clinical stability of the outcome measures included in this research over time. Furthermore, despite having carried out a single-blinded RCT following the CONSORT guidelines, it must be recognized that we used a small sample size, which hampers the detection of statistically significant differences between groups, although these may well exist. Future studies should corroborate our findings with larger sample sizes. In this line, the combination of VR with conventional rehabilitation programs presents several advantages for MS patients. First, VR systems allow repetition of goal-oriented functional tasks. Second, another of the characteristics of VR is its multi-sensorial inputs, with fundamentally visual feedback. Third, a determining factor for the implementation of VR systems in neurorehabilitation programs for MS is the motivation of the patient. Conventional therapies include repetitive and monotonous exercises that, on occasion, can cause a loss of interest on the part of patients. Fourth, an important aspect is the transfer of learning, which requires that the acquisition and training of the task be carried out in similar contexts or with characteristics that resemble the patient’s environment. For all these reasons, neurorehabilitation treatments using VR combined with conventional rehabilitation could influence the potential for brain reorganization and plasticity, allowing the learning of motor tasks [7,8].

Previously, Kim et al. [27] used the pads for the NS^®^ after transcranial direct current stimulation to study their effects on cognitive and hand function outcomes in stroke patients, and Comeras-Chueca et al. [28] reported the energy expenditure during an intervention with the NS^®^ with the RingFit^®^ combined with exercise in children with obesity. However, to our best knowledge, this is the first study that used the NS^®^ as a UL rehabilitation tool in people with MS—more specifically the right-side Joy-Con controller that includes a motion infrared sensor—in terms of grip strength, manual dexterity, functionality, quality of life, and cognitive function.

A recent systematic review showed that there is some evidence that virtual reality is effective in improving motor function in the ULs of people with MS. However, there is no clear consensus on which virtual-reality-based approaches are the most effective, or the optimum intervention duration and intensity [29]. Our results seem to be in line with this systematic review. However, although no statistically significant results were obtained in the intergroup analysis, statistical improvements were observed after the treatment in grip strength in the more affected side, the BBT for the more and the less affected side, the TMT-A, and the QuickDASH in the experimental group.

A prior study after using virtual reality in MS for UL treatment showed that this technology was warmly received in people with MS, and feedback was positive regarding both the usability of the system and the perceived sense of presence within digital environments [30]. Our results are in line with this, showing a high degree of user satisfaction with both programs in the CSQ-8. This is a key element in order to deliver optimal rehabilitation with technology [31]. Regarding the specific scale of satisfaction with the technology, patients showed a high degree of satisfaction with the experimental protocol, achieving an excellent attendance with no side effects, corroborating that this device can be used to perform movements and functional tasks with the hands in a virtual and encouraging environment for people with MS.

Feys and Straudi [31] discussed the potential of rehabilitation technology to support the achievement of key factors in motor recovery in MS, such as delivering massed practice with good movement quality, task specificity, and cognitive motor control mechanisms. In our study, we did not find significant improvements in terms of cognitive aspects in the intergroup analysis. However, we incorporated the NASA scale in our research, which is a multidimensional tool to assess the perceived workload of the tasks related to our experimental protocol. The mean score for the NASA scale was 21.62 ± 10.55 points, with the higher scores related to the Physical and Effort subscales linked to the protocol and game purposed. Future studies should study the effects of an increase in the duration (weeks) and intensity (time per treatment and number of repetitions) of the protocol, taking into account the workload of the tasks included that are assessed by the NASA scale, and incorporate other related aspects such as patients’ mood to obtain a better understanding of patients’ internal, subjective emotional state related to this technology.

Future randomized controlled trials should study the effects of our experimental protocol compared to a control group to verify our findings, incorporating measures with the technology during the experimental protocol to track the participant´s performance using this technology or other similar devices under the same infrared technology. Further, future studies should study the effects of our experimental protocol on the home treatment environment and take into account age, gender, and the EDSS score in order to conduct a deeper analysis.

This study presents several limitations. First, we used a small sample size, so future studies should be matched for age, gender, or MS severity groups. Second, a long-term follow-up was not possible because the study was conducted at an MS patient association and we had to adapt the research to the availability of the patients and the organization of the clinical centers. Third, the results cannot be generalized to all patients with MS (other EDSS scores, type of MS, and disease duration). Fourth, the sampling methods could have resulted in selection bias.

## 5. Conclusions

Our results show that an eight-week experimental protocol, after using Dr Kawashima’s Brain Training^®^ and the right-side Joy-Con controller for the NS^®^, combined with a conventional intervention, showed improvements in grip strength, coordination, fine and gross motor functions, executive functions, and upper limb functionality in the experimental group. However, no differences were observed when both groups were compared in the intergroup analysis. The addition of Brain Training^®^ for the NS^®^ for the upper limb rehabilitation did not show side effects and was rated with a high satisfaction and excellent compliance in people with MS.

## Figures and Tables

**Table 1:** Patient features.

Groups (n)	Age (Years) Mean (±Standard Deviation)	Gender	More Affected Side	Type of MS	Disease Duration (Years) Mean (±Standard Deviation)	EDSS Mean (±Standard Deviation)
Experimental group (11)	53.70 (±2.10)	7 Male 4 Female	6 Right 5 Left	RRMS: 6 SPMS: 4 PPMS: 1	20.10 (±3.39)	6.40 (±0.33)
Control group (10)	48.11 (±3.49)	2 Male 8 Female	7 Right 3 Left	RRMS: 5 SPMS: 4 PPMS: 1	17.78 (±2.68)	5.00 (±0.33)

EDSS: Kurtzke Expanded Disability Status Scale; MS: multiple sclerosis; PP-MS: primary progressive MS; RR-MS: relapsing-remitting MS; SP-MS: secondary progressive MS. Data are expressed as the mean (± standard deviation).

**Table 2:** Comparison of outcome scores (intragroup analysis).

Variable		Pre	Post	Intragroup Analysis *p*-Value
		Median (Interquartile Range)		
Grip strength more affected	Experimental group	16.00 (12.00)	17.00 (19.34)	0.033 *
	Control group	12.16 (5.83)	10.49 (11.92)	0.213
Grip strength less affected	Experimental group	17.33(22.00)	19.33 (18.66)	0.108
	Control group	20.49 (16.50)	20.00 (14.58)	0.078
BBT more affected	Experimental group	34.00 (10.00)	35.00 (19.00)	0.030 *
	Control group	36.50 (21.00)	38.00 (26.00)	0.206
BBT less affected	Experimental group	43.00 (14.00)	48.00 (15.00)	0.022 *
	Control group	44.00 (10.00)	46.00 (7.00)	0.514
NHPT more affected	Experimental group	37.68 (39.19)	36.46 (26.76)	0.346
	Control group	37.17 (39.88)	48.77 (72.56)	0.12
NHPT less affected	Experimental group	36.18 (18.00)	30.16 (18.70)	0.084
	Control group	30.44 (7.22)	31.34 (13.85)	0.400
TMT-A	Experimental group	10.46 (7.79)	6.50 (9.23)	0.012 *
	Control group	9.15 (1.55)	5.88 (4.75)	0.441
TMT-B	Experimental group	8.53 (8.93)	7.57 (15.35)	0.814
	Control group	8.61 (7.52)	7.16 (4.19)	0.173
Stroop word	Experimental group	77.00 (35.00)	82.00 (34.00)	0.789
	Control group	77.00 (17.00)	83.50 (21.00)	0.097
Stroop color	Experimental group	57.00 (24.00)	60.00 (22.00)	0.483
	Control group	56.00 (20.00)	60.50 (27.00)	0.092
Stroop word–color	Experimental group	32.00 (16.00)	30.00 (17.00)	0.646
	Control group	33.00 (18.00)	31.50 (7.00)	0.675
Stroop interference	Experimental group	0.45 (10.19)	−1.40 (17.53)	0.091
	Control group	0.67 (10.11)	−3.67 (10.24)	0.514
QuickDASH	Experimental group	36.36 (27.25)	25.00 (15.91)	0.017 *
	Control group	40.90 (19.12)	42.04 (26.71)	0.310
MSIS-29 physical score	Experimental group	55.00 (37.50)	52.50 (25.00)	0.125
	Control group	49.37 (23.44)	55.00 (25.94)	0.374
MSIS-29 psychological score	Experimental group	47.22 (47.22)	64.44 (20.00)	0.383
	Control group	24.99 (26.39)	28.60 (46.53)	0.326

BBT: Box and Block Test; NHPT: Nine Hole Peg Test; TMT: Trail Making Test; MSIS-29: Multiple Sclerosis Impact Scale. Data are expressed as the median and interquartile range. * *p* < 0.05 using the Wilcoxon test for related samples.

**Table 3:** Comparison of outcome scores between the experimental and control groups (intergroup analysis).

Variable	Experimental Group Median (Interquartile Range)		Control Group Median (Interquartile Range)		Experimental vs. Control Group	
	Pre	Post	Pre	Post	Pre *p*-Value	Post *p*-Value
Grip strength more affected	16.00 (12.00)	17.00 (19.34)	12.16 (5.83)	10.49 (11.92)	0.887	0.094
Grip strength less affected	17.33(22.00)	19.33 (18.66)	20.49 (16.50)	20.00 (14.58)	0.831	0.593
BBT more affected	34.00 (10.00)	35.00 (19.00)	36.50 (21.00)	38.00 (26.00)	0.617	0.858
BBT less affected	43.00 (14.00)	48.00 (15.00)	44.00 (10.00)	46.00 (7.00)	0.285	1.000
NHPT more affected	37.68 (39.19)	36.46 (26.76)	37.17 (39.88)	48.77 (72.56)	0.877	0.354
NHPT less affected	36.18 (18.00)	30.16 (18.70)	30.44 (7.22)	31.34 (13.85)	0.216	0.938
TMT-A	10.46 (7.79)	6.50 (9.23)	9.15 (1.55)	5.88 (4.75)	0.319	0.670
TMT-B	8.53 (8.93)	7.57 (15.35)	8.61 (7.52)	7.16 (4.19)	0.887	0.831
Stroop word	77.00 (35.00)	82.00 (34.00)	77.00 (17.00)	83.50 (21.00)	0.476	1.000
Stroop color	57.00 (24.00)	60.00 (22.00)	56.00 (20.00)	60.50 (27.00)	0.238	1.000
Stroop word–color	32.00 (16.00)	30.00 (17.00)	33.00 (18.00)	31.50 (7.00)	0.760	0.567
Stroop interference	0.45 (10.19)	−1.40 (17.53)	27.50 (24.06)	23.31 (19.27)	0.732	0.909
QuickDASH	36.36 (27.25)	25.00 (15.91)	40.90 (19.12)	42.04 (26.71)	0.859	0.080
MSIS-29 physical score	55.00 (37.50)	52.50 (25.00)	49.37 (23.44)	55.00 (25.94)	0.454	0.859
MSIS-29 psychological score	47.22 (47.22)	64.44 (20.00)	24.99 (26.39)	28.60 (46.53)	0.433	0.774

BBT: Box and Block Test; NHPT: Nine Hole Peg Test; TMT: Trail Making Test; MSIS-29: Multiple Sclerosis Impact Scale. Data are expressed as the median and interquartile range.

## Data Availability

No new data were created or analyzed in this study. Data sharing is not applicable to this article.

## Supplementary Materials

The following supporting information can be downloaded at: https://www.mdpi.com/article/10.3390/jcm11123261/s1, Figure S1: Experimental protocol.
