# Supplementary material for: Nintendo Switch Joy-Cons’ Infrared Motion Camera Sensor for Training Manual Dexterity in People with Multiple Sclerosis: A Randomized Controlled Trial
Source: J Clin Med. 2022 Jun 7;11(12):3261. doi: 10.3390/jcm11123261 (PMC9224824; doi:10.3390/jcm11123261)
Supplement: Supplementary file 1 [file jcm-11-03261-s001.zip › jcm-1732629-supplementary.pdf]

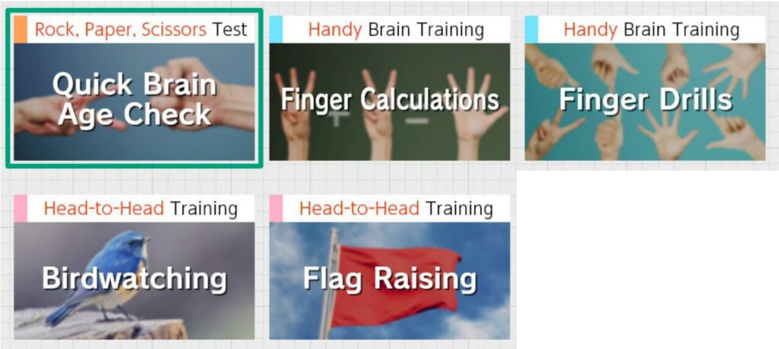

Flag Raising **Finger Drills**

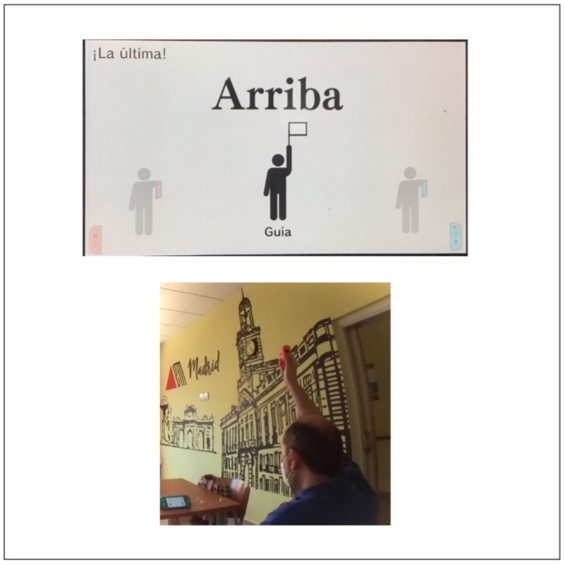

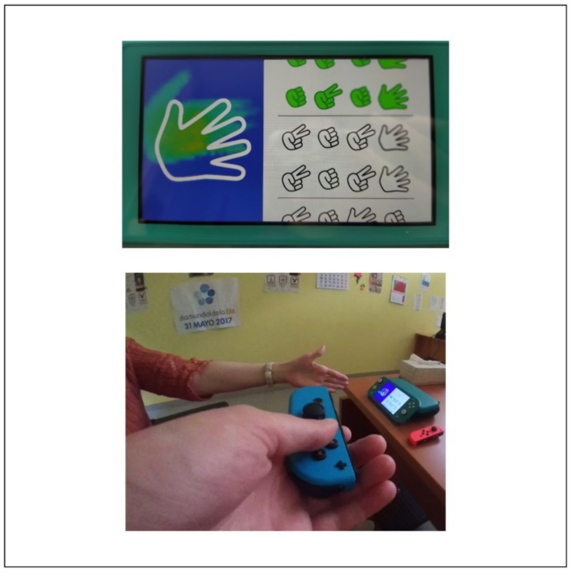

Birdwatching **Finger Calculations**

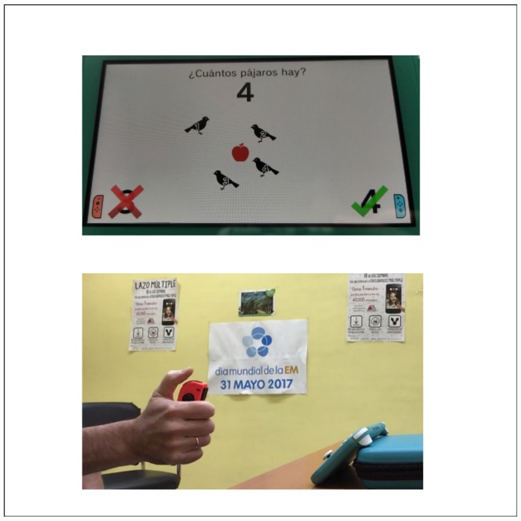

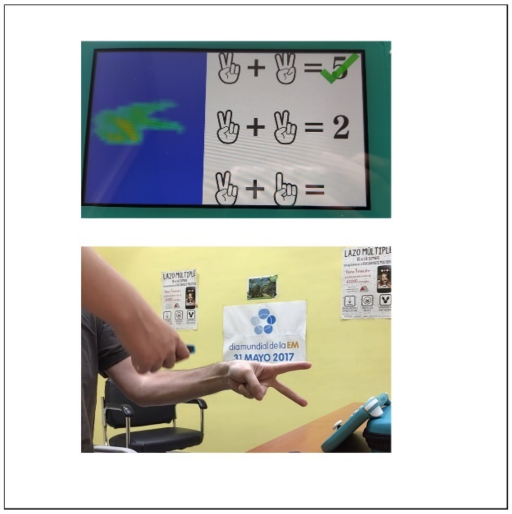

Rock-Paper-Scissors game

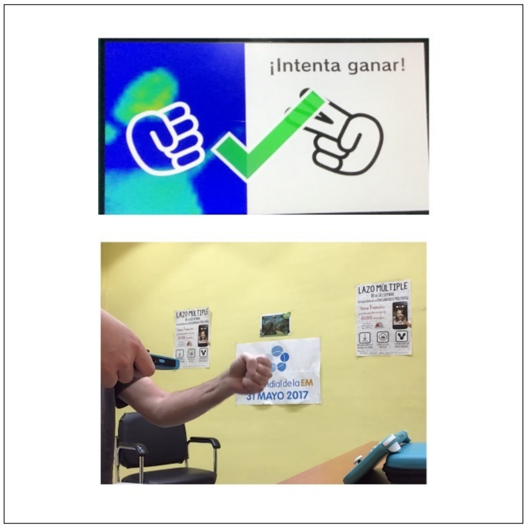

Figure S1. Experimental protocol.
